# Proximity induced signatures of elusive Bose metal phase in topological insulator- superconductor junction

**DOI:** 10.1038/s41598-025-11256-8

**Published:** 2025-07-23

**Authors:** Reena Yadav, Mandeep Kaur, M. P. Saravanan, Sudhir Husale

**Affiliations:** 1 Academy of Scientific and Innovative Research (AcSIR), Ghaziabad, 201002 India; 2 National Physical Laboratory, Council of Scientific and Industrial Research, Dr. K S Krishnan Road, New Delhi, 110012 India; 3 Low Temperature Laboratory, UGC-DAE Consortium for Scientific Research, University Campus, Khandwa Road, Indore, 452001 India

**Keywords:** Superconductivity, Bose metal phase, Topological insulators, Bismuth telluride, Low temperature transport, Nanodevice, Materials science, Nanoscience and technology, Physics

## Abstract

The quantum metal state (QMS) occurring between the superconductor and insulator transition is often considered a Bose metal phase (BMP) whose understanding remains elusive and has been under debate even though studied for decades. To observe the BMP one needs to disrupt the phase coherence and search it into 2D amorphous, disorder, defective or nanoengineered superconducting materials. Superconductor -Topological insulator (SC-TI) junctions host the exotic nature of quasiparticles and are expected to show 2D superconductivity. Here, for the first time, we harness exotic SC-TI junctions investigating signatures of BMP and report proximity induced low temperature transport through superconductor-bismuth telluride (SC-Bi_2_Te_3_) nanosheet junctions. Transport data reveals superconducting effects in the nanosheets and the existence of the resistive metallic state with reentrant nature. We analysed the data to show the appearance of different quantum states. For longer junction lengths (1.1 & 0.78 μm), temperature-dependent resistive humps having similar peak heights and widths in the magnetoresistance (MR) curves were observed which were suppressed for smaller junction length (310 nm). The hump signatures in MR curves and scaling analysis of the data indicate the appearance of BMP. Our results suggest that SC-TI junctions exhibiting partial superconductivity are necessary to witness a peculiar metallic state resembling a BMP.

**Supplementary Information:**

The online version contains supplementary material available at 10.1038/s41598-025-11256-8.

## Introduction

Recently, the intriguing properties of TI and SC junctions have attracted considerable attention as a promising system for realizing topological superconductivity and exploring exotic quantum states relevant for quantum computation devices^1–3^. TI possesses robust transport properties and transport through surface state has been observed by various groups at low temperatures^4–8^. TI materials like Bi_2_Te_3_, Bi_2_Se_3_ etc. are not superconducting but they show superconducting effects due to the injection of quasiparticles when they come in contact with superconducting material^9–16^. Theoretically, it is proposed that SC-TI junctions can host coveted Majorana fermions, useful for quantum computation^17–20^. Experimentally, it has been found that superconducting pairs can be formed on the surface states of TIs and signatures of topological superconductivity have been demonstrated in heterostructure based junctions^21,22^.

Induction of Cooper pairs in the system of topological surface states (TSS) that show a robust nature and no back tunnelling of electrons, may lead to a new superconducting phase due to the formation of unconventional symmetry of pairing^23^. Due to injection of quasiparticles, many interesting quantum effects have been reported and some of them are still elusive to understand convincingly e.g. re-entrant transition^24,25^, mysterious peaks^26^, negative magnetoresistance^27^ etc. Studies on Sn-Bi_2_Se_3_ junction reported several conductance anomalies below the T_c_ (superconducting transition temperature) of Sn including the presence of temperature dependent zero bias peak^23^. On the other hand, understanding superconductivity in the presence of a strong magnetic field in thin films or 2D nanosheets is one of the fundamental problems. A numerous experiments have been performed to resolve the puzzle of a superconductor to insulator transition arising either due to Cooper pair breaking or loss of long-range coherence^28,29^.

Interestingly, an anomalous metal phase with resistivity saturation to a finite value has been observed in 2D superconducting systems due to disorders or the presence of the magnetic field^29,30^. Previously the quantum metallic transition has been observed in 2D disordered^31^ and clean (or low disorder) systems^30,32^. In disordered systems, Cooper pairs remain together but become localized due to disorder and interactions. This often referred as Bose metallic phase where the system exhibits a universal resistance h/4e² (≈ 6.45 kΩ) nearby the point of transition^31^. The work done on amorphous films of InOx or MoGe supports MPA Fisher’s theory^33^. Whereas the clean system focuses on pair-breaking, phase or quantum fluctuations. Here the Cooper pairs are not localized but they move freely in the absence of external factors such as magnetic field. Interestingly, it has been observed that clean 2D systems like ZrNCl and other clean materials^34,35^ also show finite resistances even at very low temperatures and referred as quantum or anomalous metal which is not predicated by Fisher’s theory. This indicates the need of more theoretical work to explain both the systems and overall, the observation of QMS in various systems remains elusive^36^.

Nanosheets of TI are an excellent system for observing proximity-based 2D superconductivity. Nanosheets offer a high surface-to-volume ratio, minimal or no contribution of bulk effects and an easy system for Fermi-level tuning. Earlier, numerous studies on the SC-TI interface have observed that the long range proximity effects are of diffusive nature^13,22,37^. It is expected that a magnetic field can disturb the phase coherence of the system and can drive the system into a frustrated or disordered nature where the quantum metal state can be observed. The work on 2D superconducting thin films and devices showed an intermediate QMS in the phase diagram whose origin is still under investigation and has triggered more experiments^30^.

It has been observed that, when TI placed in contact with a conventional S-wave superconductor, the proximity effect can induce exotic superconducting phenomena such as surface state can host p wave like pairing, topological superconductivity and due to the interplay between spin momentum locking & superconductivity, Majorana bound states can occur at interfaces^20^.The proximity based unconventional superconducting phenomena consistent with a sign changing order parameter was observed in Bi_2_Te_3_ flakes contacted with conventional aluminium SC electrodes^13^. In our recent results on Bi_2_Se_3_ flakes and W electrodes junctions, we observed anomalies in MR curves^38^ but such anomalies were not present when we contacted the TIs flakes with the normal metal^39^. Strong proximity based superconducting effects have been reported in thin flakes of Bi_2_Te_3_ and supercurrents were observed over a longer inter electrode distance of about 3.5 μm which indicates that the Bi_2_Te_3_ is a good material to study proximity induced superconductivity^14^. This triggers us to investigate further and fabricate junctions of superconductor and TI- Bi_2_Te_3_ nanosheet.

Here we investigate the junction length (JL) dependent proximity induced superconductivity in SC-TI junction devices. The magnetic field drives the transport into an unexpected intermediate resistive state which appears below the transition of superconducting electrodes for longer JLs. We observed new signatures which show the hump formation in MR and resistance-temperature (RT) curves indicating the formation of a peculiar quantum transition. Lowering the temperature, superconductivity recovers and peculiar quantum transition disappears. Scaling analysis suggests the signatures of Bose metal phase. Here our experiments, first time show that there is no requirement for disorder thin films or high-quality crystals, the resistive metallic state can also be evolved in proximity-induced partial superconducting devices made of SC-TI interface. Further, this work will be a new entrant to the list of materials where intermediate metallic phases are reported so far.

## Experimental

**Fig. 1:**
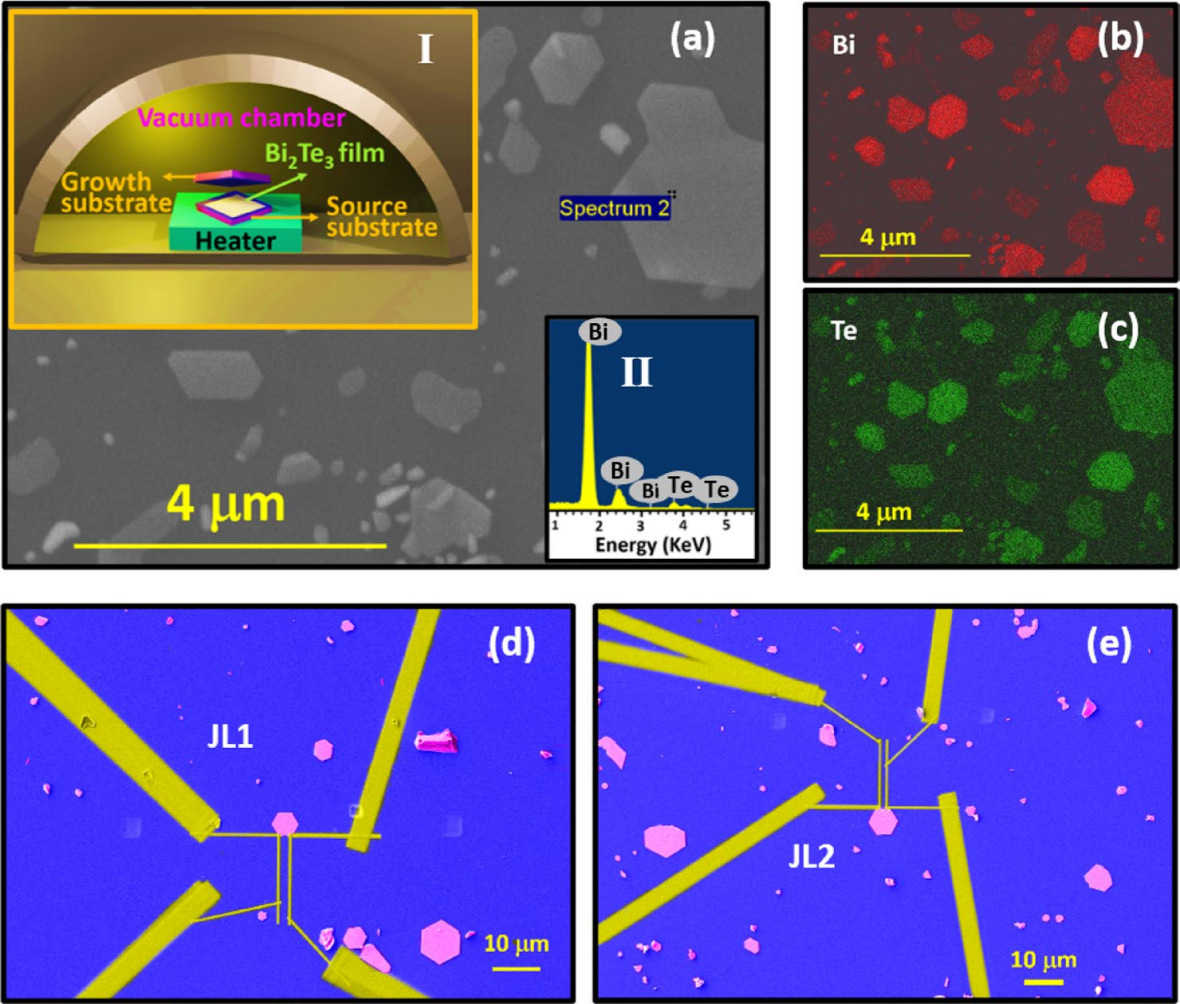
Synthesis, material characterizations and device fabrication: **a**) shows the FESEM image of Bi_2_Te_3_ nanosheets grown on Si_3_N_4_/Si substrate. Inset I is the schematic of nanosheet growth using confined thin film melting method and inset II is the EDS spectra of a particular selected nanosheet. **b&c**) are elemental mapping images of bismuth and tellurium respectively, performed using EDS. **d&e**) display the FESEM images of Bi_2_Te_3_ nanosheets contacted by superconducting W electrodes.

The hexagonal Bi_2_Te_3_ nanosheets used for proximity studies were grown on Si_3_N_4_/Si or SiO_2_/Si substrate by using the confined thin film method as reported earlier^40,41^ and schematic for the same is shown in the inset I of Fig. 1a. The nanosheets were thoroughly characterized for their morphological, elemental composition and crystallographic quality using FESEM (field emission scanning electron microscope), EDS (energy dispersive spectroscopy), Raman and HRTEM (high resolution transmission electron microscopy) techniques. Figure 1a shows the growth of various Bi_2_Te_3_ nanosheets on Si_3_N_4_/Si substrate. The EDS spectra and mapping were carried out to know the atomic ratio of elements (inset II) and the presence of elements Bi (Fig. 1b) & Te (Fig. 1c). In order to analyze the chemical components and crystal structures, nanosheets were examined by HRTEM. low & high magnification TEM picture, SAED pattern and Raman spectra of synthesized Bi_2_Te_3_ nanosheets are shown in Supplementary Sect. 1.

Further, the nanosheets were contacted using focused ion beam assisted deposition of W superconducting electrodes. During deposition utmost care was taken for proper placement of nanoscale electrical pads on the nanosheets. These nanoscale W pads were finally connected to big (500 μm x 500 μm) sputter-deposited Au pads. The final device structure looks like a single Josephson junction-type configuration as shown in Fig. 1d and e and in Supplementary Sect. 2. Here two contacts of voltage and current are separated by the device’s junction length (JL1&JL2) of about 1.1 μm and 780 nm respectively. During low-temperature characterization, a magnetic field was applied perpendicular to the sample (Bi_₂_Te_₃_ nanosheets), and the excitation current was approximately 100 nA. Low-temperature resistance measurements, from 300 K down to 2 K, were performed using Quantum Design’s Physical Property Measurement System (PPMS) equipped with a 16 T magnet. Note that, in studies of proximity-induced superconductivity, the choice of electrical measurement geometry—2-probe, 3-probe, or 4-probe—significantly impacts data interpretation. A 2-probe configuration, where current and voltage are measured across the same electrodes, is simple but includes contact and interface resistances, often leading to over estimation of the induced gap or spurious features due to electrode effects^42^. In contrast, a 3-probe setup, with separate current and voltage leads near the SC/normal interface, better isolates interface phenomena such as Andreev reflection and can be used to estimate the local proximity-induced superconducting gap^23^. However, some residual contact resistance may remain. The 4-probe technique, which uses distinct current and voltage pairs, eliminates contact resistance and can be considered for studying long-range superconducting correlations and zero-resistance states in the normal region, thus providing strong evidence of phase-coherent transport^43^. However, 4-probe measurements do not directly probe the interface and must be interpreted cautiously, especially in disordered or phase-inhomogeneous systems, where the induced superconducting gap may not be uniform. Combining different geometries provides complementary insights into interface physics versus extended coherence in proximity systems.

## Results

**Fig. 2:**
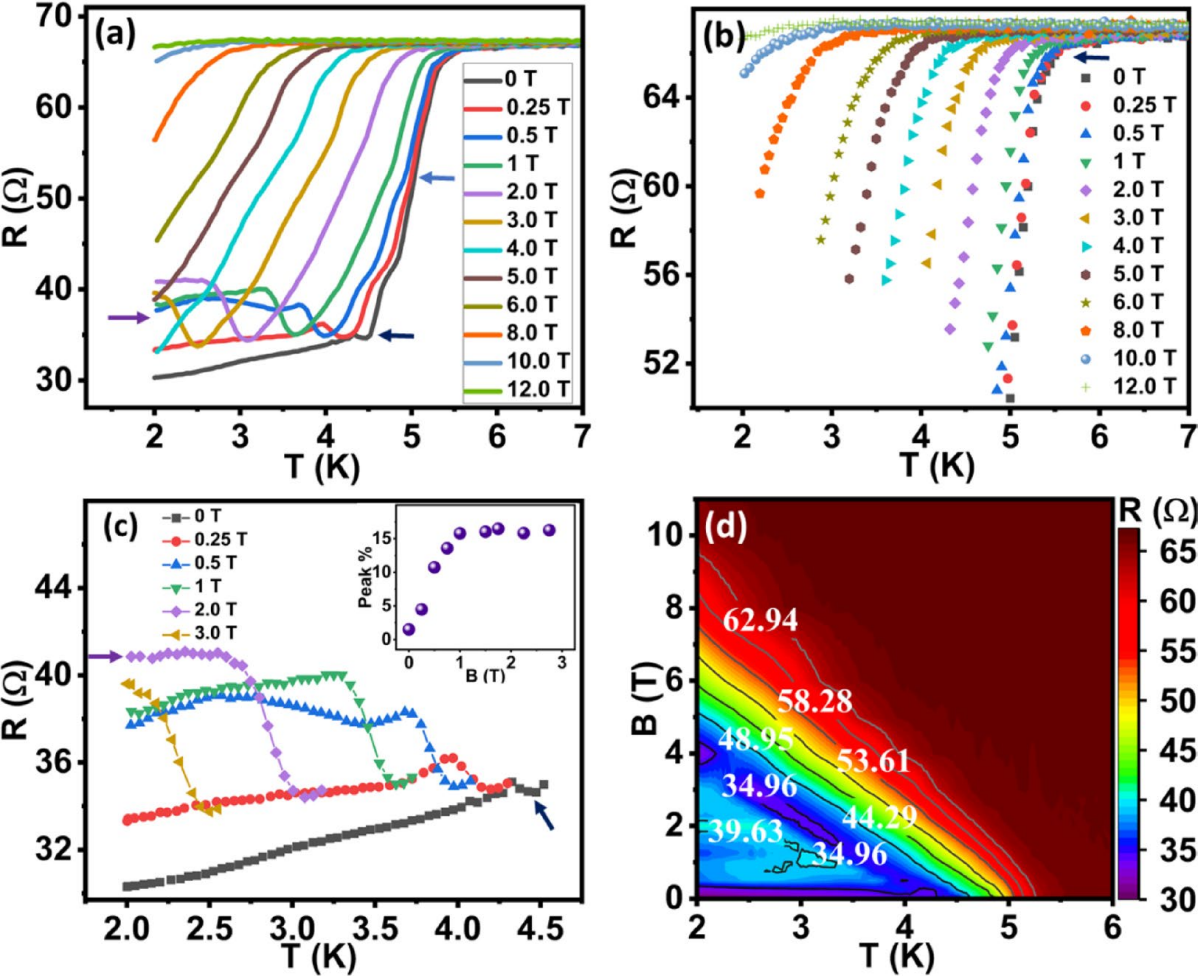
Analysis of RTH curves for JL1 (1.1 μm) device: **a**) Magnetic field dependent RT curves display 3-step superconducting development and drop in resistance. **b&c**) represent the 2 different parts of RTH curves. **d**) displays the phase diagram of RTH curves.

**Fig. 3:**
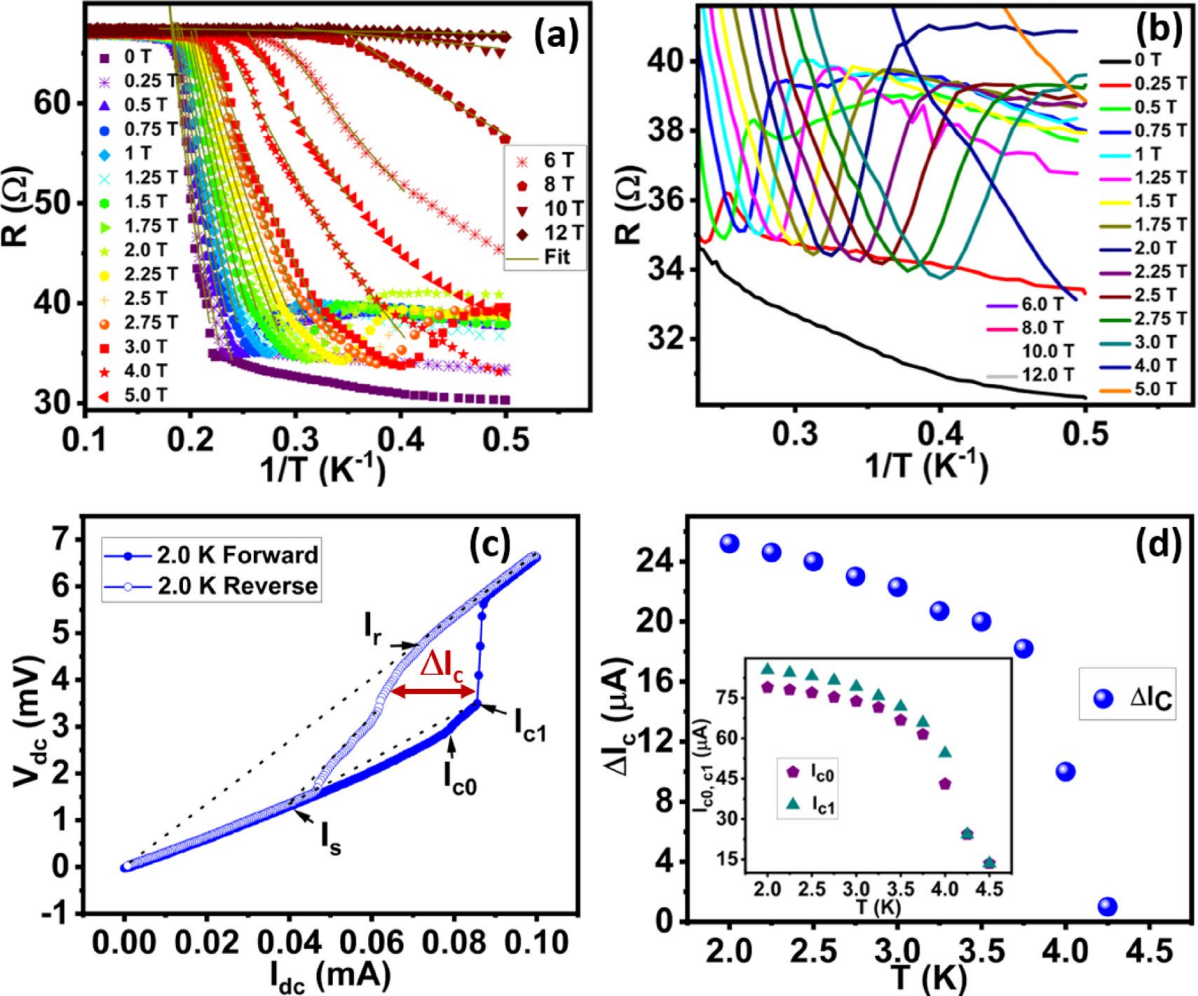
Estimation of activation energy and IV characteristics of the JL1 (1.1 μm) device: **a**) shows the Arrhenius plot (R vs. 1/T) at different magnetic fields. **b**) the zoom portion of Figure **(a)** displays an upturn and resistance saturation plateaus. **c**) IV characteristics of the junction and **d**) hysteresis current observed in the IV curves and inset shows the temperature dependence of critical currents (I_c0_ and I_c1_).

**Fig. 4:**
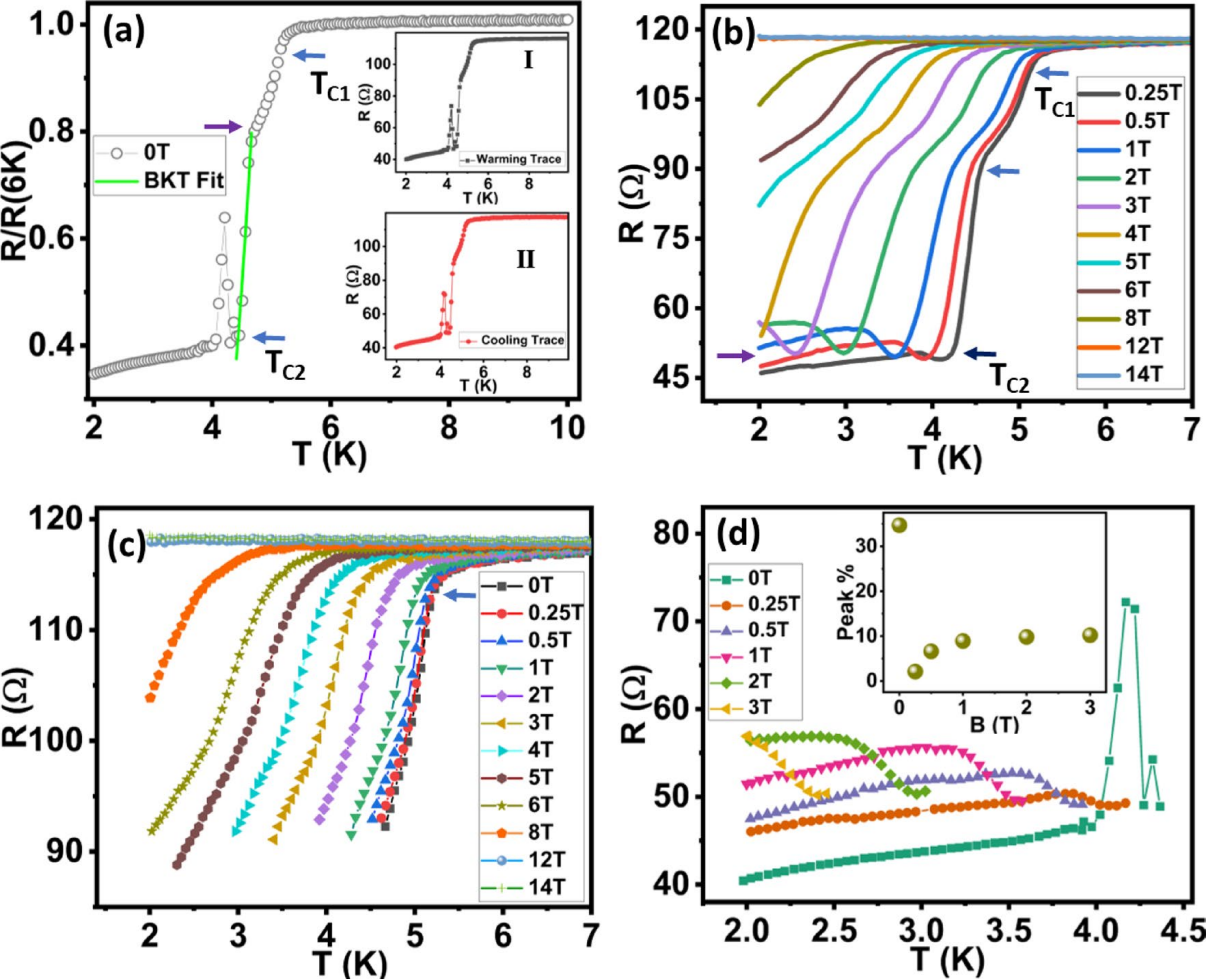
Analysis of RTH curves for JL2 (780 nm) device: **a**) RT curve at 0 magnetic field displays 2 step superconducting transition and a mysterious peak. **b**) shows magnetic field dependent RTH curves. **c-d**) represent 2 different parts of RTH curves and inset in Figure (**d**) is the % rise of the upturn transition.

**Fig. 5:**
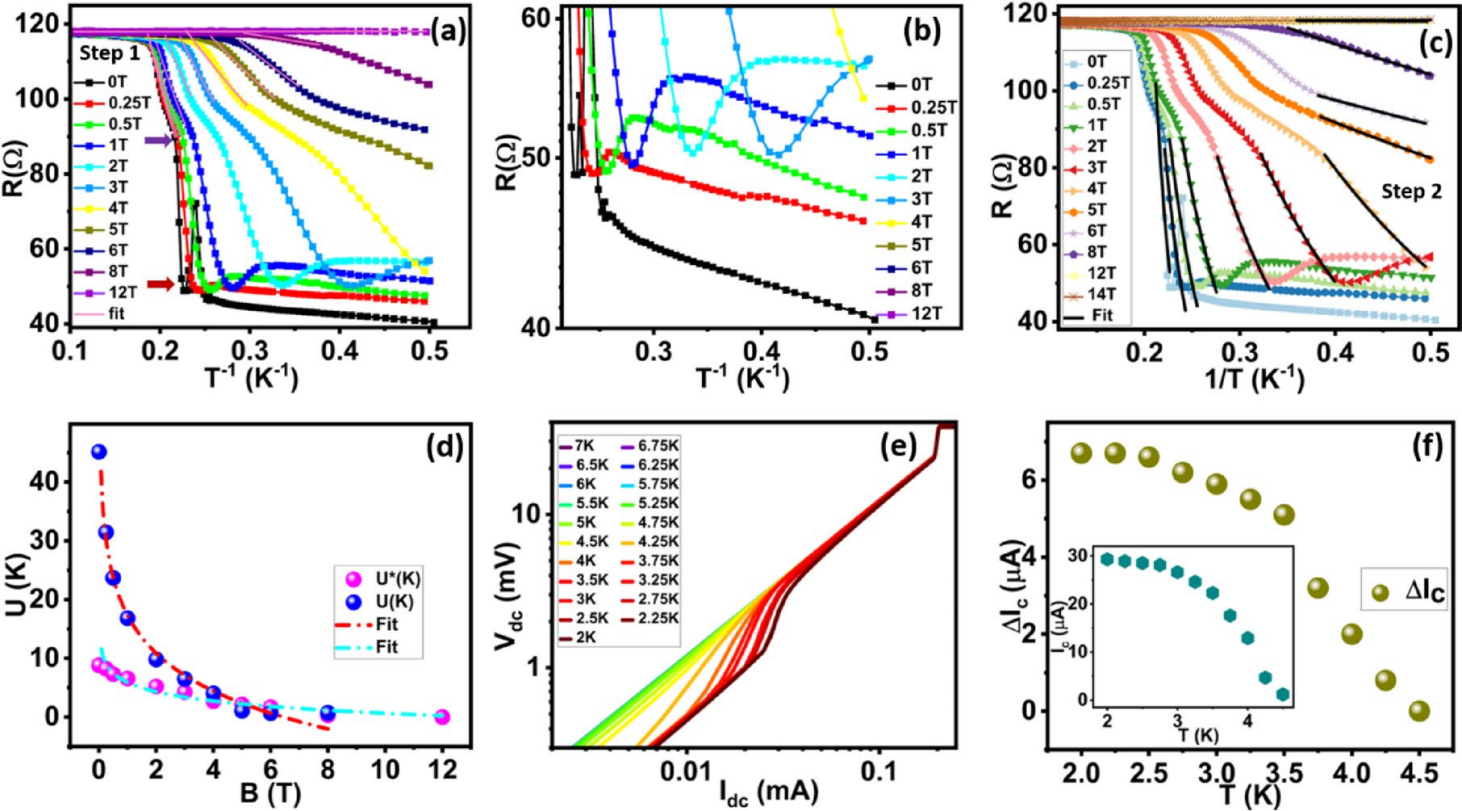
Estimation of activation energy and IV characteristics of the JL2 device: **a**) Arrhenius plot (R vs. 1/T) at different magnetic fields shows the thermally active regions and solid lines show the linear fits for the step 1 transition **b**) zoom image of the resistance saturation regime in the Arrhenius plot. The zoom portion of Figure **(a)** displays an upturn and resistance saturation plateau. **c**) Arrhenius plot exhibiting thermally active regions for the step 2 transition (solid lines) **d**) estimation of energy barrier from linear fits for steps 1 and 2. (e) Temperature dependent IV characteristics of the junction (JL2 Device). (f) represents the hysteresis current in the IV curves and estimation of ΔI_c_. Inset represents the temperature dependence of critical current.

**Fig. 6:**
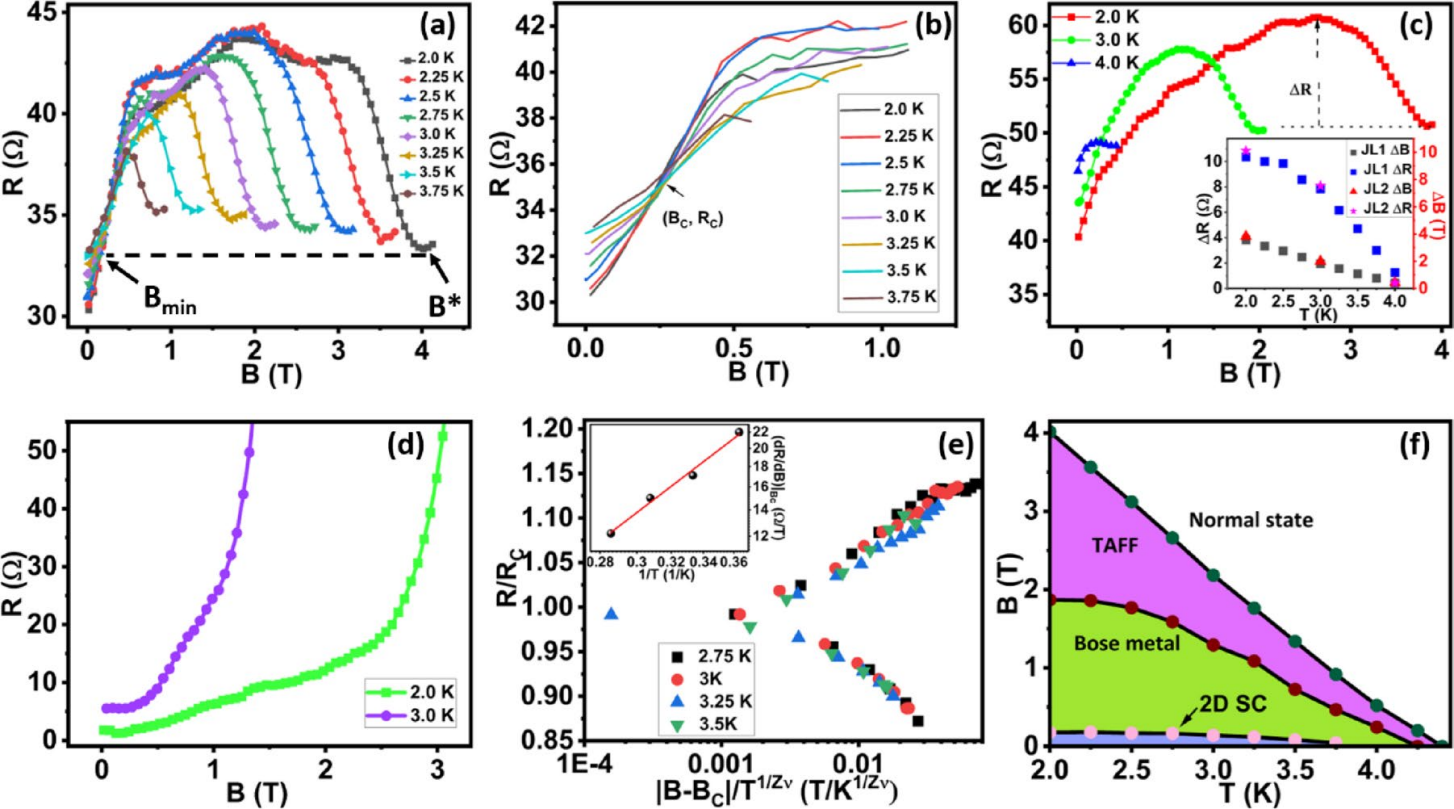
Signatures of Bose Metal Phase. **a**) Temperature dependent MR curves of the JL1 (1.1 μm) device shown upto 4 T. **b**) Magnified data of Figure a) depicting Superconductor to Bose metal quantum transition. **c**) MR curves plotted for the JL2 (780 nm) device, show the reproducibility of the hump curves. Inset shows the change in resistance and magnetic field (ΔR = peak of the hump- minimum fall of the resistance from the peak position, ΔB = width of the hump, B^*^ – B_min_). **d**) MR curves for JL3 (310 nm) device, show fading of hump structures. **e**) Scaling analysis of MR curves of JL1 (1.1 μm) device. Inset represents the estimation of exponent zv ∼ 0.42. **f**) Phase diagram depicting Bose metal state estimated from the Figure a) data. Critical field (denoted by B* in Fig. 6 (a)) is the field at which R starts to increase with decreasing field. Peak (maxima) of resistance is the cutoff between thermally assisted flux flow (TAFF) and the Bose metal state. B_min_ which defines 2D SC state is calculated by the intersection of MR curves with the black dotted line.

It is known that FIB-deposited W strips/nanowires exhibit a sharp superconducting transition at T_c_ ⁓ 5 K^44,45^. Our earlier results also demonstrate that W is a highly reproducible superconducting material, suitable for proximity effect or photon detection studies^46,47^. Compared to other techniques, FIB deposition is a simple, controlled and direct deposition technique. Here we explain the data of three devices having junction lengths (JLs) of about 1.1 μm (JL1), 780 nm (JL2) and 310 nm (JL3) in Figs. 2, 3, 4, 5 and 6. First, we describe the proximity induced transport through a JL1 device. This device’s junction resistance (the normal state resistance at 10 K) is around 67 ohms, indicating a good interface to observe proximity effects. This is also supported by the BTK (Blonder-Tinkham-Klapwijk) theory^48^ where the conductance (Y) of a normal metal –superconductor junction can be written as

1 $$\:\text{Y}\:\left(\text{Z},\text{T}\:\right)=\:\left(1+{Z}^{2}\right){\int\:}_{-\infty\:}^{\infty\:}\frac{\partial\:f\:}{\partial\:E}\left[2A\left(E\right)+C\left(E\right)+D\left(E\right)\right]\:\:\:\:\:\:\:\:\:\:\:\:\:\:\:\:\:\:\:\:\:\:\:\:\:\:\:$$

Where Z describes the barrier strength of the junction. A(E), C(E), and D(E) are functions defined in the BTK theory. At T = 0, Eq. 1 can be simplified as

2 $$\:\:\:\:\:\:\:\:\:\:\:\:\:\:\:\text{Y}=\frac{2(1+{\text{Z}}^{2})}{{(1+2{\text{Z}}^{2})}^{2}}\:\:\:\:\:\:\:\:\:\:\:\:\:\:\:\:\:\:\:\:\:\:\:\:\:\:\:\:\:\:\:\:\:\:\:\:\:\:\:\:\:\:\:\:\:\:\:\:\:\:\:\:\:\:\:\:\:\:\:\:\:\:\:\:\:\:\:\:\:\:\:\:\:\:\:\:$$

By using this formula and a saturated conductance value of about 1.78 (Supplementary Sect. 2 Fig c), we estimate the Z value of JL1 (1.1 μm) device ∼ 0.2. Since this barrier value is close to the transparent limit (i.e., Z = 0) and not in the tunneling limit (Z >> 1), hence we believe that our devices can show a strong proximity effect.

Figure 2a shows the temperature-dependent resistance (RT) graph of JL1 (1.1 μm) device (black curve) in the absence of a magnetic field. The decrease in resistance at ∼ 5 K indicates the superconducting transition of tungsten electrodes. The resistance sharply drops up to 52 Ω, then there is a gradual decrease in resistance down to 35 Ω which indicates the injection of Cooper pairs from superconducting electrodes (SC) into the Bi_2_Te_3_ nanosheets. Further, a decrease in temperature shows a small upturn behaviour in resistance over a small temperature window (4.2–4.47 K). Beyond this, the drop in resistance shows only a minor decrease as a function of the decrease in the temperature. Measurements were repeated under different magnetic fields as shown in Fig. 2a. For a better understanding, the data have been divided into three sub-parts, indicated by the blue (Fig. 2b), black (Supplementary Sect. 3 Fig a) and purple (Fig. 2c) arrows in Fig. 2a.

The arrow in Fig. 2b shows the onset T_c_ (∼ 5.4 K) of the nanosheet and superconducting electrode junction which is the superconducting transition of FIB-deposited tungsten electrode. The drop in resistance from 67 Ω to 55 Ω is sharp (black squares and red circles) and onset T_c_ is shifted as we increase the magnetic field indicating the superconducting nature of the sample. A further drop in resistance from 55 Ω to 35 Ω depicts a gradual decrease in resistance as a function of temperature with the slanting nature of the curves as shown in Supplementary Sect. 3 Fig a. Such a decrease in resistance is due to the injection of Cooper pairs in the nanosheet and coupling phase coherence increases with decrease in temperature. The curves were shifted to lower temperature with an increase in the magnetic field. A small knee-like feature was noticed for low magnetic field < 0.5 T but disappeared for the high fields. The slow diffusion of Cooper pairs in the normal metals / semiconductors has been observed in the long range proximity based superconducting effects. It is known that proximity induced transition exhibits two or three steps and has previously been reported in Nb-Sb_2_Te_3_ nanoribbon - Nb junctions^49^ and superconducting islands placed on normal metal^50^.

A decrease in resistance < 35 ohm, has witnessed a small upturn (black curve in Fig. 2c) in the resistance value as shown by the black arrow. This upturn in resistance vanished with a decrease in temperature. After this resistive upturn, the resistance of the sample decreased very slowly (black curve from 4.3 K to 2 K). Interestingly, with an increase in field, an upturn in resistance was becoming more prominent and the sample clearly shows a resistive state in superconducting transport. The inset in Fig. 2c shows the resistive upturn peak rises to 15% at 1.25 T and then the resistance saturates for higher fields. We observed that the sample was becoming more resistive with an increase in the magnetic field and the reappearance of the superconducting behaviour of the sample was not visible with our instrument measurement limit. The resistance saturation at lower temperatures depends on the applied magnetic field. At 2 T, the RT curve (purple colour Fig. 2c) shows metallic behaviour (purple arrow) with a resistance saturation plateau. The decrease in resistance is negligible for the measurement from 2.7 to 2 K. This signature indicates the formation of a peculiar QMS in the sample at low temperatures. Such a peculiar QMS has emerged in pristine 2D superconductor^51^ disorder amorphous InOx films^33^NbSe_2_^30^, and amorphous films of MoGe^52^ and Ta^29^. Many experimental and theoretical work have been performed to understand the origin of dissipation in this QMS^30,36^^53^–^55^.

From the RTH (resistance as a function of temperature in the presence of magnetic field) curves, we estimated the critical field ($\:{B}_{c2}\left(T\right)$) for 40% resistance drop as shown in Supplementary Sect. 3 Fig c. The GL fit equation as shown below was used to know the $\:{B}_{c2}\left(T\right)$ of JL1 (1.1 μm) device.

3 $$\:{\text{B}}_{\text{c}2}\left(\text{T}\right)={\text{B}}_{\text{c}2}\left(0\text{}\right)\left[1-{\left(\frac{\text{T}}{{\text{T}}_{\text{c}}}\right)}^{2}\right]\:\:\:\:\:\:\:\:\:\:\:\:\:\:\:\:\:\:\:\:\:\:\:\:\:\:\:\:\:\:\:\:\:\:\:\:\:\:\:\:\:\:\:\:\:\:\:$$

where $\:{B}_{c2}\left(0\right)$ is the upper critical field at temperature 0K and T_c_ is the critical temperature. Here we estimated upper critical field $\:{B}_{c2}\left(0\right)$ ∼ 5.9 T, T_c_ ∼ 4.6 K and a coherence length of about 7.47 nm which are very close to the reported values of FIB deposited W strips or wires^45^. Note that the B_c__2_ values depend on the electrode material used to induce the superconducting effects in the sample. Here we used FIB deposited W as an electrode to induce superconductivity which has B_c__2_ of about 9T^45^. Previously B_c__2_ values due to the proximity effect in TI were reported from a few hundred mT to few T. The work done by Wang et al. (2015) showed a proximity-induced superconducting transition and measured upper critical field B_c__2_ about 0.2 T^56^. Whereas He et al. studied interface superconductivity in the heterostructure of Bi_2_Te_3_ /FeTe and indicated B_c__2_ value more than 14 T^12^. Other reports on the materials SrBi_2_Se_4_, Co nanowire and TI Bi_0.91_Sb_0.09_ used GL fit either to report B_c__2_ values about 2.1 T^57^ and 11 T^58^ or to estimate coherence length^59^. The B_c__2_ values of the devices JL1 (1.1 µm) & JL2 (780 nm) were estimated using GL theory (Supplementary Sect. 3 Fig c&d). The B_c2_ values were found in the range ~ 6 T but estimating it using GL theory is not appropriate here because we observed it due to proximity based induced superconductivity in topological insulator system which need further experimental and theoretical understanding.

The phase diagram of RTH curves is shown in the Fig. 2d. It clearly shows the reappearance of the high resistive phases at low field (< 3 T) and low temperature (< 3.5 K). The lines in the graph exhibit the value of resistance change. For high field > 5 T and temperatures above 4.6 K, a monotonically increase in the resistance has been observed but below this field and temperature limit, a decrease in resistance exhibited the evolution of multiple resistive patches as shown by the change in colour. A resistance displays saturation type behaviour below 0.3 T & 3.5 K temperature as shown by the purple colour.

Figure 3a shows the estimation of activation energy from the Arrhenius plot (R versus I/T data) using the following relation.

4 $$\:R\:\propto\:\text{exp}\left(-\frac{U\left(B\right)}{T}\right)\:\:\:\:\:\:\:\:\:\:\:\:\:\:\:\:\:\:\:\:\:\:\:\:\:\:\:\:\:\:\:\:\:\:\:\:\:\:\:\:\:\:\:\:\:\:\:\:\:\:\:\:\:\:\:\:\:\:\:\:\:\:\:$$

where U (B) is the activation energy. It is also called a pair dissociation energy.

All the curves initially start from a normal state resistance, indicating the system is in the normal state. Due to the diffusion of Cooper pairs from the W electrode into the sample, resistance shows a linear or sublinear fall dominated by the dissipation due to thermally driven vortex - antivortex pair formation where binding and unbinding of pairs play an important role. Due to the longer junction length (1.1 μm) and measurement limitations, the curves do not show perfect superconductivity. The 55% drop in resistance is visible for 0 T field and the drop has almost vanished for fields > 12 T. The Lower part of the graph for higher fields (> 0.5 T) shows a small upturn and saturation in resistance. For clear visualization, we plot it separately in the Fig. 3b. The saturating resistance plateau is noticeable for field 2 T. Previously such strange resistive metallic state was also referred as Bose metal state^30,60^. The values of linear fits were used to estimate the energy barrier U, needed to create a vortex-antivortex pair and its dependency on the magnetic field is plotted in supplementary Sect. 3 Fig e. Here we estimated U_0_ using the following pair dissociation energy (U(B)) versus magnetic field fit relation.

5 $$\:U\:\left(B\right)={U}_{0}\text{ln}\left(\frac{{B}_{0}}{B}\right)\:\:\:\:\:\:\:\:\:\:\:\:\:\:\:\:\:\:\:\:\:\:\:\:\:\:\:\:\:\:\:\:\:\:\:\:\:\:\:\:\:\:\:\:\:\:\:\:\:\:\:\:\:\:\:\:\:\:\:\:\:\:$$

The fit values yield characteristics vortex unbinding energy U_0_ = 4.08 K and B_0_ = 11.25 T which is slightly deviated from our estimated value of B_c2_. Deviation of B_c2_ values from B_0_ has been observed previously in NbSe_2_ flakes where Bose / quantum metal state was studied^30,51^ .

Figure 3c shows the I-V measurements of JL1 (1.1 μm) device. The forward and reverse biased curves measured at 2 K are shown by filled and empty blue dots respectively which clearly show the presence of hysteresis indicating the superconducting nature of the sample. The transition from superconducting to normal metallic state does not occur in a single step at 2 K and an intermediate resistive step appears that merge at the excess current, *I*_*s*_. The retrapping current (*I*_*r*_) is the current that flows through a sample as it passes from a resistive to a superconducting state in a reverse bias direction. *I*_*c0*_ and *I*_*c1*_ are the critical currents, defined as the onset of finite voltage from the minimum voltage level and the intermediate resistive step, respectively. The measurements were repeated for different temperatures. The forward bias curves and reverse bias curves are shown in Supplementary Sect. 4 Fig a&b. The hysteresis current (ΔI_c_) between forward and reverse bias curves as a function of temperature is shown in Fig. 3d and the critical currents I_c0_ and I_c1_ are shown in the inset of Fig. 3d. Both the curves show a decrease in current values with an increase in temperature.

Now we show the proximity-induced superconducting effects and evolution of quantum metal state for JL2 device (junction length ∼ 780 nm, Fig. 4). The device image is shown in Fig. 1e. The resistance starts to drop at ∼ 5.3 K which is the onset superconducting transition temperature of the W electrodes. A drop in R witnesses two steps as shown by the arrows in Fig. 4a. At temperature 4.2 K, a sudden upturn resistance peak was noticed. Interestingly, this mysterious peak was reproducible during cooling and warming cycles (Inset I and II) but vanished when the field > 0.25 T was applied (Fig. 4b) Observation of resistance/conductance anomalies below the T_c_ of W is consistent with the earlier observation reported on topological insulator Bi_2_Se_3_ and superconductor Sn junction where the formation of chiral superconducting phase was observed^23^. Earlier work by Wang et al. observed a mysterious sharp resistance peak near the T_c_ of the W electrodes and the intensity of the peak was diminishing with the applied perpendicular field^26^. In our case, the peak vanished even with the small field and was not present for the JL1 (1.1 μm) and JL3 (310 nm) devices. Moreover, we can not rule out the presence of disorder, high quality TI-SC interface in our sample because we use FIB based method to fabricate these samples which has limitations for making very narrow JJs. The origin of this peak could be due to the junction interface resistance which needs more investigations.

After this peak, the device shows a gradual decrease in resistance from 4 K to 2 K. Magnetic field dependent RT curves show low temperature shifting of device’s onset T_c1_ and T_c2_ values. The two-step transition and resistance upturn features are visible in RTH curves as shown in Fig. 4b. For a clear view of the data, we split RTH curves into three parts as shown in Figs. 4c&d and Supplementary Sect. 3 Fig b.

Similar to the longer junction device JL1 (1.1 μm), this device also shows an upturn, 65% drop in resistance and saturation behaviour due to proximity induced superconductivity (Fig. 4d). The inset shows an upturn percentage of resistance from its maximum drop. Considering our measurement limits, we observed a maximum 10% upturn increase in the junction resistance. The partial superconductivity gets disrupted due to applied magnetic field in the perpendicular direction, upturn in resistance exhibits an increase of ∼ 7 Ω which is 3 orders less than the pair quantum resistance $\:\frac{h}{{4e}^{2}}$ = 6.4 kΩ. This in agreement with the good sample quality where many systems show QMP resistance can appear lower than the normal metal state resistance^30^. To estimate the critical field for JL2 (780 nm) device, we used GL fit as shown in Eq. 3. The fit gives the value of B_c2_ (0) of about 5.18 T and coherence length ∼ 8 nm (Supplementary Sect. 3 Fig d). The earlier work on Bi_2_Se_3_ topological insulator also observed the decrease in critical field and this could be due to interplay of the spin polarized current of the surface state and Cooper pairs of the superconducting electrodes^61^.

To study the temperature-dependent activated resistance behaviour of the JL2 (780 nm) device, we again use Arrhenius plotting of the data (Fig. 5a). The curves show two steps drop in resistance from the onset T_c1_. The first drop in R originates due to W electrodes becoming superconducting and 2nd drop is more resistive compared to the first drop. Similar to JL1 (1.1 μm) device, JL2 (780 nm) also shows upturn and resistance saturation behaviour for high field curves (Fig. 5b). The curve at 2T shows a resistance saturation plateau and no gradual decrease in resistance indicates the evolution of a strange resistive quantum metal state.

To estimate the vortex-antivortex pair dissociation energy U(B), we use Eq. (4) to fit the linear portion of the first drop and second drop as shown in Fig. 5(a&c) respectively. From fit, we obtain the values of energy barrier U whose dependency on the magnetic field is shown in Fig. 5(d). Pink and blue circles show the energy barrier value for the first and second drops which are denoted by U*(K) and U (K). Here values of U_0_, B_0_ are 2.28 K, 13.36 T for first drop and 9.3 K, 6.45 T for the second drop respectively and calculated by using Eq. (5). Here, B_0_ for 2nd drop is slightly deviated from estimated B_c2_ while B_0_ for 1st drop is ∼ 2.5 times of B_c2_.

Figure 5e shows the IV characteristics of JL2 (780 nm) device from 2 K to 7 K at zero magnetic field. The critical current was defined as the onset of finite voltage from the minimum voltage level as shown in the inset of Fig. 5f. Below the superconducting transition temperature, measurement of IV curves shows hysteresis during forward and reverse sweep directions. This indicates the existence of vortex-antivortex pairs in the JL2 (780 nm) device. We observed that critical current I_c_ and ΔI_c_ reduce to zero near the normal state (Fig. 5f).

These results indicate that our devices operate as phase-slip dominated weak-link Josephson junctions. The observed hysteresis in the current–voltage (I-V) characteristics and the multistep resistive transitions are consistent with transport through a fluctuating superconducting region, as typically seen in long superconductor–normal metal–superconductor (SNS) junctions. We estimated the characteristic voltage product I_c_R_N_ for the four-probe device 1 to be approximately 0.207 mV (Supplementary Sect. 5). For the two-probe devices JL1 and JL2, I_c_R_N_ values ranged from 0.5 to 1.6 mV at 4.25 K, and from 3 to 5 mV at 2 K, respectively. These I_c_R_N_ values confirm the proximity-induced superconductivity in the junctions. Importantly, all resistance–temperature (RT) and magnetoresistance (MR) measurements were performed using a low excitation current of 100 nA—well below the critical current. This ensured that the voltage drop remained within the superconducting sub-gap regime (i.e., below the superconducting energy gap Δ).

Nanostructures of topological insulators show surface state transport and has been reported in many systems. It is anticipated that TIs interfaced with the SC electrodes would exhibit 2D superconducting transport signatures (Supplementary Sect. 4 Fig d). Even though our JL2 (780 nm) device shows partial superconductivity, we have 2D superconducting transport features. It is known that Cooper pairs from the superconductor are not supposed to persist over a longer junction length but experimentally proximity based superconducting effects showed the survival of Cooper pairs as long as ≤ 1 µm^62^ and partial superconducting effects for JLs > 1µm^26^. To confirm that the observed proximity effect arises from topological surface states (TSS) in Bi_₂_Te_₃_, we estimated the nanosheet thickness and carrier density using structural and spectroscopic characterizations. High-resolution TEM and SAED analysis confirm the rhombohedral phase with the c-axis perpendicular to the nanosheet plane, while Raman spectroscopy reveals characteristic A_₁g_^¹^, E_g_^²^, and A_₁g_^²^ phonon modes, along with IR-active A_₂u_^¹^ and A_₂u_^²^ peaks—signatures consistent with few-quintuple-layer Bi_₂_Te_₃_ and prior studies on ~ 27QL films^63^. AFM measurements indicate an average thickness below 50 nm (~ 45–50 QL), placing our samples in the quasi-2D regime where TSS dominate transport. Using the measured normal-state resistance (~ 67 Ω), device geometry, and a reported mobility of ~ 630 cm²/Vs, we estimate a carrier density of ~ 3.5 × 10¹⁹ cm⁻³ via the Drude model, consistent with surface-dominated transport in Bi_₂_Te_₃_ nanosheets^64^. These values suggest that the Fermi level resides near or within the Dirac cone of the TSS, providing favorable conditions for the emergence of surface-mediated proximity-induced superconductivity observed in our devices.

Figure 6 indicates the different signatures of the quantum metal state. Figure 6a&b represent the MR curves of JL1 (1.1 μm) device plotted up to field 4 T and 1.1 T respectively. Figure 6a displays the B_min_ field required for a superconductor to strange metal transition which is clearly shown in the zoom data (Fig. 6b). At field ~ 0.26 T, a critical point or quantum phase transition of crossover has been observed. A similar crossover has been reported in thin films of superconductor indicating the features of the Bose metal phase^33,65^. The field dependent resistance increases linearly and shows a saturation plateau followed by a decrease in resistance (we termed it a ‘hump’). The length of the hump (ΔB) is temperature dependent and it increases as we decrease the temperature. Similarly, the hump height (ΔR) is also temperature dependent but saturates at lower temperatures. Surprisingly both JL1 (1.1 μm) and JL2 (780 nm) devices show similar hump height (ΔR) and hump length (ΔB) indicating no dependency on the junction lengths for these devices (inset Fig. 6c). A hump in MR curves is clearly visible in Figs. 6a&c but missing for JL3 (310 nm) device (Fig. 6d) which shows > 95% drop in resistance indicating proximity based strong superconducting nature of the device compared to the partial superconducting devices - JL1 (1.1 μm) and JL2 (780 nm). For JL3 (310 nm) device, fewer grains could make favourable conditions for interland phase coherence. Note that for JL3 (310 nm) device, we have done measurements up to 2 K and do not see a hump, maybe this device needs a lower temperature (mK range) or to destroy the phase coherence to see such effects. Observation of hump indicating Bose metal phase is consistent with the earlier report on amorphous InO_x_^33^ films and impedance based studies performed using NbSe_2_ nanolayers^51^.

In Fig. 6e we show the temperature scaling analysis investigating the nature of metallic transition as discussed in other studies of 2D tantalum films^29^. Near magnetic field induced transition or crossing point of all isotherms (B_c_), the resistance can be rescaled as $\:R(B,T){\propto\:R}_{c}F\left(\left|B-{B}_{c}\right|{T}^{-\frac{1}{z\nu}}\right)$, where *F(x)* is a universal scaling function with *F(0) = 1*. By adjusting the field as $\:\left(\left|B-{B}_{c}\right|{T}^{-\frac{1}{z\nu}}\right)$, all the curves may be collapsed into a single curve.

The inset in Fig. 6e shows the plot of $\:{\left(\frac{dR}{dB}\right)}_{{B}_{c}}$ vs. 1/T. The inverse slope of this plot gives the value of zν. We estimate the critical exponent zν of about 0.42 for the JL1 (1.1 μm) device. B_c_ is determined from the magnetic field induced transition observed at 0.26 T as shown in the Fig. 6b. The experimental characteristic of this state shows power law dependence on the correlation length $\xi\:\propto\:{\left|B-{B}_{c}\right|}^{-\nu}$ and correlation time $\:\tau\:\propto\:{\xi}^{z}\propto\:{\left|B-{B}_{c}\right|}^{-z\nu}$ coordinates. From the scaling analysis, we observed that Fig. 6e shows the collapsing of all the curves on a single curve.

The quantum metal state also characterized using the MR data scaling method where the field-induced resistance in Bose metallic phase can be described using the relation $\:R\propto\:{\left(B-{B}_{c0}\right)}^{2\nu}$ where ν is the exponent of superfluid correlation length and B_c0_ is the critical field observed for superconductor to Bose metal transition (Supplementary Sect. 4 Fig c). In MR curves, we observed two linear regimes, one is at a low field before reaching the peak of the hump and other one is at a high field which starts once the hump peak reaches to a minimum B value. At low field, MR increases linearly and then saturates. Note that, long junction lengths show partial superconducting effects. Supplementary Sect. 4 Fig c shows linear scaling of high field MR data of JL1 (1.1 μm) device fitted using the relation $\:R\:\sim\:{\left(B-{B}_{c0}\right)}^{2\nu}$. The obtained 2ν values plotted as a function of T in the inset show that exponent values 2ν decreases as we increase the temperature. The power law with unity exponent value for MoGe films^66^1.61 for NbSe_2_ layers^30^ has already been reported suggesting the quantum tunnelling of fermionized vortices.

The Fig. 6f shows the phase diagram of the Bose metal phase as a function of magnetic field and temperature. The Bose metallic phase occupies a significant portion of the phase diagram. The Bose metal resistance increases to a peak which helps to define the cutoff point between Bose Metal phase and thermally activated flux flow (TAFF). Using the values of B* and B_min_ as shown in the Fig. 6(a) and supplementary Sect. 6, we plot the phase diagram for metallic phase for junction lengths JL1 (1.1 μm) as shown in Fig. 6f. Overall the experimental analysis shows the emergence of a quantum metal state in partial superconducting topological insulator-based junctions.

## Discussion

Here we discuss the superconductivity, re-entrant resistance and possible origins of a peculiar metallic phase (hump) observed in our experiments. Note that devices JL1 (1.1 μm), JL2 (780 nm), and JL3 (310 nm) do not show a transition to a zero-resistance state in the RT curve. The devices made here represent two probe geometry and to rule out the influence of the W electrodes in the observed proximity effect, we carried out 4 probe measurements (Supplementary Sect. 5). The hump like features in MR curves are clearly visible which is consistent with the two probe data reported in Fig. 6a. Proximity induced superconductivity depends on the junction length, superconducting material’s coherence length, interface quality, mobility of the host material, diffusion length etc. Device JL3 (310 nm) does not show any re-entrant behaviour and more than 95% superconducting state was observed (RT and MR curves are shown in the Supplementary Sect. 5 Fig d&e). Many reports also mention the observation of proximity-based superconductivity in topological insulator material. Some of them observed partial superconducting effects, while others reported long-range superconductivity^14,61^. It has been observed that the proximity-based superconductivity does not show sharp transitions and resistance decreases gradually^49,50^. Longer JL favours the diffusive motion of Cooper pairs arising from the quantum phase fluctuations. It is expected that a magnetic field can disturb the phase coherence of the system and can drive the system into a frustrated or disordered nature where the quantum metal state can be observed. From Fig. 6a-d, we can say that the suppression of QMS depends on the junction length.

Further, for the first time, we report the re-entrant resistive state which is more pronounced in the presence of high field, lower temperature and long-range proximity. In the presence of an external magnetic field (< lower critical field), mobile vortices (quantized flux lines) may be generating dissipation in the sample increasing the resistance. The destruction of superconductivity and the appearance of resistance could be due to phase fluctuations of the order parameters. The re-entrant behavior has been observed several times in the past, either described as mysterious peaks or seen in systems such as ferromagnet–superconductor junctions, where it occurs due to spin accumulation effects^26^. Similarly the upturn in resistance was observed in proximity based devices e.g. Bi_2_Se_3_ with different SC electrodes Al, In and W as they become superconducting^61^. The upturn in resistance was also observed in Nb-Bi_2_Te_3_ hybrid structures due to the low transparency at the junction interface resulting in suppression of Andreev reflection and energy barrier effect induced by the superconducting electrodes^9^. Using BKT analysis here we observed that device JL2 (780 nm) exhibit two-dimensional superconductivity with signatures of broad transitions (Supplementary Sect. 4 Fig d). Note that here we report proximity effect by observing only partial superconductivity, resistance was not observed to zero and these devices represent two probe measurement geometry which is different from a simple 4-probe measurement. The IV curves shown here in Fig. 5e might be consisting of the affection from the interfacial effects such as the Andreev reflections, disorders or superconducting W electrode etc. which limit data analysis using the BKT transitions.

It is important to note that the IV measurements presented in Figs. 3c and 5e were performed in two-probe configuration. As such, the measured voltages include contributions not only from the partially proximitized Bi_₂_Te_₃_ nanosheet but also from the superconducting W electrodes and interface resistances. This leads to apparent voltages exceeding the extracted induced gap (~ 0.2 meV, Supplementary Sect. 5), which was estimated using four-probe conductance analysis. Nevertheless, the features observed in the IV curves—hysteresis, intermediate resistive states, and nonlinearity—are consistent with weak-link Josephson junction behavior in proximity systems. Assuming the junction as a dissipative weak-link, the extracted I_c_R_N_ value from 4 probe measurements support the proximity effect interpretation and indicate the interpretation of a proximity-induced quantum metallic state in the Bi_₂_Te_₃_ nanosheets. Thus to understand the local interface effects, such as Andreev processes or localized gap features accurately 3 probe measurements may be considered.

The observed resistance in RT graphs (Fig. 2a) is composed of resistance of the superconducting electrode (can be considered as negligible near T_c_), resistance of the interface and resistance of the Bi_2_Te_3_ nanosheet. This is equivalent to the transport measurement through TI (parallel circuit between surface and bulk) and a series circuit between two TI – superconductor interface and two superconducting W electrodes. Note that JL1 (1.1 μm) and JL2 (780 nm) devices show incomplete proximity coupling just below the T_c_ of the W electrodes. This could be due to long channel distance and superconducting correlations are not coherent. In addition there could be some finite interface transparency considering disorder/defects/damage implanted during device fabrication, suppression of the gap near the interface & phase fluctuations in the weakly coupled surface states which could give rise resistance below the T_c_. The tunneling probability of quasiparticles is more near the T_c_ of W due to small superconducting gap which enhances the chances of incomplete Andreev reflections indicating the extra interface resistance. Further the freezing of bulk state transport through TI at low temperature may increase the total resistance. The slight decrease in resistance value at lower temperature indicates decay in quasiparticle population resulting less dissipation, growing superconducting gap improving the coherence length and surface states are more coherently active through proximity effects. The resistance saturation observed at low temperature (Fig. 2c) indicates the emergence of quantum metal phase which we studied here using scaling function. Note that theoretically, it would be controversial whether such a quantum metal state can really exist in the superconducting proximity effect and need more understanding in the future. Furthermore, the finite resistance observed below T_c_ in devices JL1 and JL2 can be attributed to the presence of phase-slip lines (PSLs) or weak-link behavior within the junction. This interpretation is supported by the measured I_c_R_N_ values aligning closely with the estimated superconducting gap, and the hysteretic IV behavior indicating underdamped Josephson dynamics. Previous work using FIB-deposited W on topological insulators has reported similar PSL formation due to spatial inhomogeneity at the interface^59^. These weak-link effects, combined with the influence of magnetic field and disorder, provide a natural basis for the emergence of a Bose metal-like quantum metallic phase in our SC–TI devices.

Recent work on NbSe_2_ metal by Banerjee Abhishek et al. extracted the Bose metallic phase by subtracting resistance values from RT curves of low impedance and high impedance conditions used in their experiments^51^. Depending on the dissipation the Bose metallic phase curves show three steps, mainly initial increase, saturation and decrease in resistance. This work reports the observation of the Bose metal phase in a clean environment. In a dissipative environment, the Bose metal phase vanished as soon as the system was coupled to a nondissipative bath. In particular, a temperature-dependent hump-like structure in the presence of a parallel magnetic field was also reported in the nanostrips of MoGe samples^52^. The occurrence of intermediate resistive state was observed due to unbinding of twisted vortex and the decrease in resistance was attributed to a straightening of the vortex lines and higher vortex concentration at higher field^52^. Interesting to note that signatures of hump observed in MR curves resemble our data but the system used in our case is completely different suggesting the universality of hump signature to probe further as a Bose Metal phase.

The observation of quantum metal phase depends on the development of superconducting fluctuations in the devices JL1 (1.1 μm) & JL2 (780 nm) studied here. It is known that the proximity effect has pair amplitude but no pair potential, the superconducting fluctuations may develop in the topological insulator–superconductor (TI–SC) junctions due to the presence of topological surface states and interplay of reduced dimensionality, disorder at the interface or inherent defects in device fabrication process etc. The Bi_2_Te_3_ hexagonal nanosheets used here are of 2D nature and thickness is less than 50 nm whereas smallest distance between two superconducting contacts is about 310 nm. Thus smaller dimensions indicate the possibility of confinement effect. The earlier work where FIB technique was used to deposit W electrodes and reported the formation of tungsten (W) clusters on TI (Bi_0.91_Sb_0.09_). These clusters forms the Josephson Junctions indicating the natural formation of phase-slip lines (PSLs) and confinement effects^59^. Here we state that formation of such ultra nanoclusters is uncontrolled, random & inherent process in FIB based metal deposition. Hence the area of about 50 nm^2^ nearby the deposited electrode material must have W clusters that may help in the natural formation of Josephson Junctions or disordered weak links indicating the role of spatial and temporal fluctuations in the pair amplitude enough for the origin of superconducting functions. Further it has been observed that in TI-based Josephson junctions, the supercurrent can exhibit sample-specific mesoscopic fluctuations due to interference effects among multiple paths of Cooper pair transport on the surface states. Here the Josephson current fluctuations due to disorder and quantum coherence show that the fluctuations are signatures of non-uniform proximity based superconductivity^67^.

Earlier Galitski et al. reported the formation of vortex metal where interactions of electrically neutral spinons and field induced vortices result in a broad magnetoresitance peak at low temperature^68^. Phillips et al. and other researchers have proposed the formation of Bose metal phase which is a gapless, nonsuperfluid state that can be observed in the bosons interacting 2D system where uncondensed Cooper pairs and vortices in presence magnetic field play an important role^36,69,70^. The resistance in Bose metal state depends on the Josephson coupling of neighbouring domains (q) and effective dissipation factor (n) by the relation $\:=\frac{h}{4{e}^{2}}\:\frac{3{m}^{4}}{qn}$. This tells that the resistivity is reciprocally dependent on the product of *qn*. Smaller JL predicts stronger coupling of Cooper pairs between the domains and strong superconducting state without dissipation can be achieved as seen in Fig. 6d. Due to the influence of perpendicular magnetic field, motion of vortices gets disrupted. Further, we have observed the field induced resistance follows the linear scaling and collapsing of all the curves in a single curve (Fig. 6e). Note that, all of our devices are exposed to the same fabrication and instrument measurement conditions hence we do not see problems with external noise or filters used during the measurements. The above analysis and observed signatures of the MR curves suggest that the Bose Metal Phase is evolving in the SC-TI junction and may be investigated further.

## Conclusions

Our results show that W Superconducting electrodes can be used to observe proximity based superconducting effects in the topological insulator nanosheets. Depending on the JL, either partial or strong superconductivity can be achieved. The low temperature transport shows existence of a strange metallic state exhibiting a hump like structure which is more pronounced in large JLs and vanishes with decrease in JL. First time, the proximity induced superconducting devices show the signatures of quantum metal phase indicates the Bose metal phase in our samples. We believe that topological insulator based nanosheet hosts robust system to observe 2D superconductivity triggering more work for further confirmation of quantum metal phase. The observed proximity based QMS in our samples (TI and SC junction) is not addressed by any existing theories and triggers further future theoretical and experimental work.

## Methods

The information about Bi_2_Te_3_ nanosheets is shown in the supplementary Sect. 7.

## Electronic supplementary material

Below is the link to the electronic supplementary material.

Supplementary Material 1

## Data Availability

The datasets used and/or analysed during the current study available from the corresponding author on reasonable request.
