# Supplementary material for: Proximity induced signatures of elusive Bose metal phase in topological insulator- superconductor junction
Source: Sci Rep. 2025 Jul 23;15:26688. doi: 10.1038/s41598-025-11256-8 (PMC12283962; doi:10.1038/s41598-025-11256-8)
Supplement: Supplementary file 1 — Supplementary Material 1 [file 41598_2025_11256_MOESM1_ESM.pdf]

Supplementary Material

Proximity induced signatures of elusive Bose Metal Phase in topological insulator-superconductor junction

Reena Yadav[1,2], Mandeep Kaur[2], M. P. Saravanan[3] & Sudhir Husale[1,2]*

[1] Academy of Scientific and Innovative Research (AcSIR), Ghaziabad-201002, India

[2] National Physical Laboratory, Council of Scientific and Industrial Research, Dr. K S Krishnan Road, New Delhi-110012, India

[3] Low Temperature Laboratory, UGC-DAE Consortium for Scientific Research, University Campus, Khandwa Road, Indore 452001, India

** Corresponding author*

Email : husalesc@nplindia.res.in

1. Supplementary Section 1. Structural and Spectroscopic Characterization of Bi_2Te_3 Nanosheets

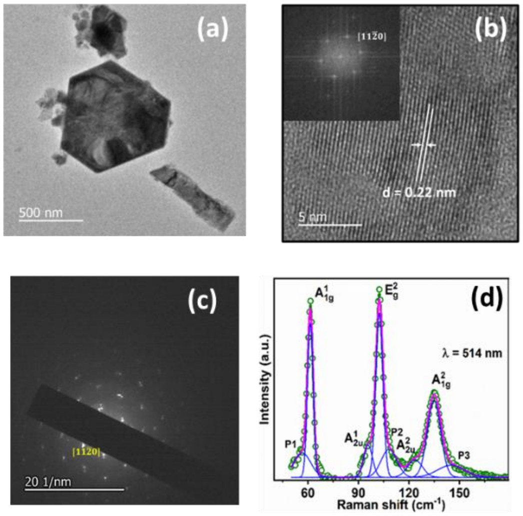

Supplementary Figure S1: (a) Low magnification TEM image of synthesized nanosheet of Bi_2Te_3 , (b) HRTEM characterization and the inset shows the corresponding FFT pattern, (c) SAED pattern of Bi_2Te_3 nanosheet, (d) Raman spectra of the Bi_2Te_3 nanosheet

High resolution Transmission Electron Microscopy analysis of Bi_2Te_3 Nanosheets- A thin hexagonal nanosheets like morphology with sharp edges was found in the TEM image with edge dimensions of about 215-565 nm (a). An atomic-scale or HRTEM lattice image of fringes is shown in figure b. The anticipated lattice fringes with a lattice spacing of 0.22 nm are observed in the HRTEM image, consistent with the $(11\bar{2}0)$ planes of Bi_2Te_3. The corresponding FFT pattern is displayed in the inset of figure b. This pattern revealed a crystalline nature oriented

along [0001] facets displaying a hexagonal symmetry of the rhombohedral phase of Bi_2Te_3 (space group: R-3m) with lattice constant a = b = 4.39 A^0, c = 30.48 A^0 (JCPDS card no. 150863). The SAED pattern (Fig c) shows a collection of significant planes of the rhombohedral crystal structure of $(11\bar{2}0)$ with an interplanar spacing of 0.22 nm. Similar to our work, nanoparticles having $(11\bar{1}0)$ as the side surface, (0001) as the top and bottom surface grown along the $(11\bar{2}0)$ direction have been reported in previous studies[1-3]. The Raman spectroscopic experiment was performed at excitation wavelength λ = 514 nm to investigate the quality of hexagon nanosheets as shown in figure (d). We observed three Raman active optical phonon peaks corresponding to A_{1g}^1 (~61.6 cm^{-1}), E_g^2 (~102.4 cm^{-1}) and A_{1g}^2 (~ 134.9 cm^{-1}) and two infra-red active modes A_{2u}^1 (~95 cm^{-1}) and A_{2u}^2 (~123.5 cm^{-1}). A vibrational mode A_{2u}^1 is described as surface phonon mode (SPM) which occurs only in nano-sized materials[4]. In addition to characteristic peaks, we observed P1, P2 and P3 peaks as marked in figure d. The peak P2 is caused by size effect of nano structures and Infra-red active modes of Bi_2Te_3 whereas, peaks similar to P1 and P3 are already observed in the thin film of Bi_2Te_3[5]. The observed Raman data is consistent with the previously reported work on Bi_2Te_3 nanostructures and thin films[4-6].

2. Supplementary Section 2. Device Fabrication and electrical characterization

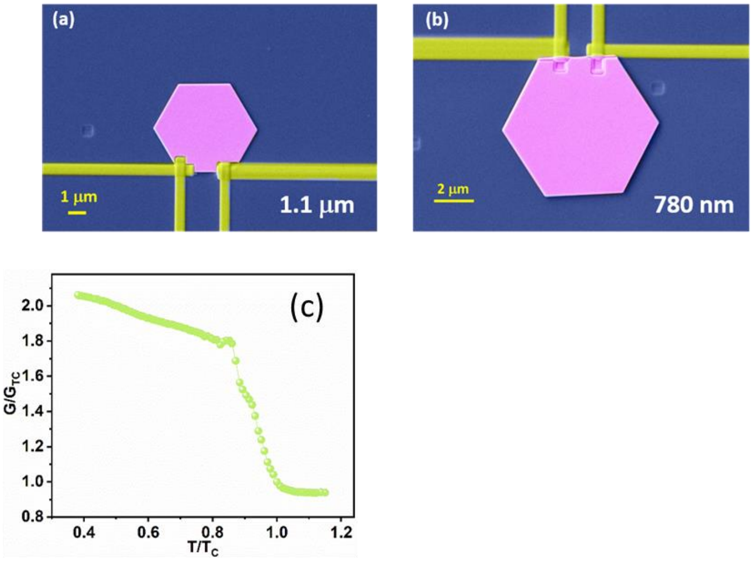

Figure S2: (a&b) False colored FESEM images of devices JL1 and JL2 respectively, (b) conductance versus temperature plot of JL1 device

3. Supplementary Section 3. Temperature dependent selected transport data

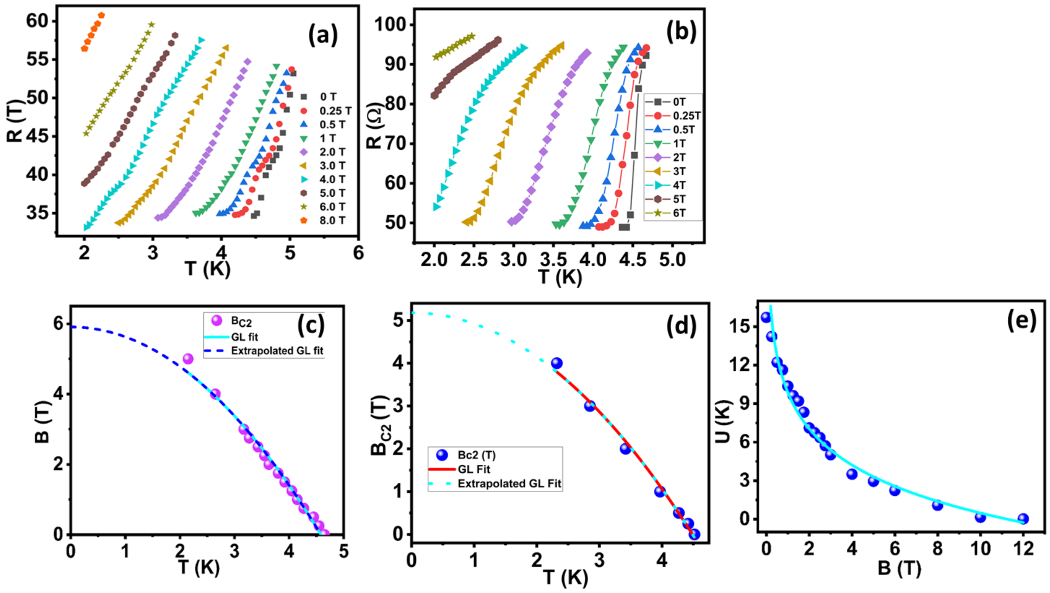

Figure S3: (a) Selected magnified RTH data of JL1 device , (b) Selected magnified RTH data of JL2 device , (c&d) Upper critical field Bc_2 extracted by fitting R–T–H curves using GL theory, for JL1 & JL2 devices respectively, (e) the estimated energy barrier U(B) as a function of magnetic field shows dissociation of vortex–antivortex pairs and its dependency on the magnetic field.

4. Supplementary Section 4. IV and Scaling Analysis

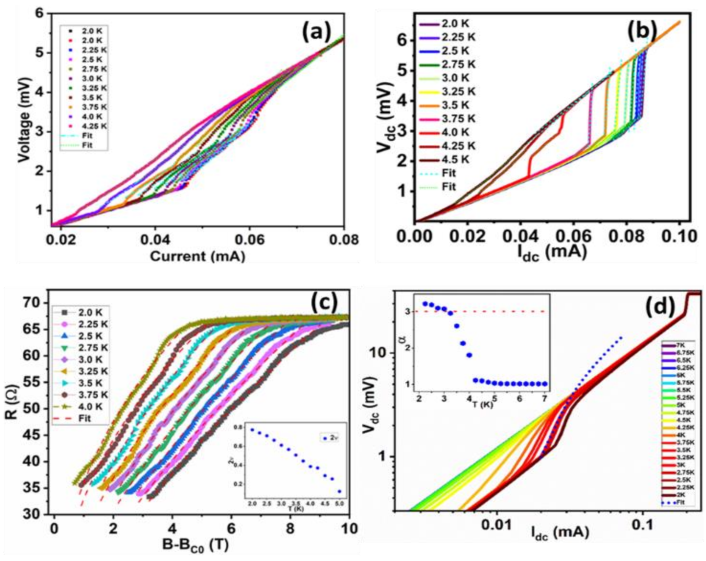

Figure S4: (a &b) Temperature-dependent IVs. The forward and reverse bias IV curves of JL1 device respectively, (c) Linear scaling of high-field magnetoresistance (MR) data analyzed via field-induced Bose metal scaling relations for JL1 device, (d) Power-law IV analysis of JL2 device Temperature dependent IV characteristics of the junction (JL2 Device). The extraction of 'α' using power law fitting $V \propto I^{\alpha(T)}$. Inset represents α (exponent) versus temperature data.

5. Supplementary Section S5: Four-probe transport measurements and transport data of JL3 device

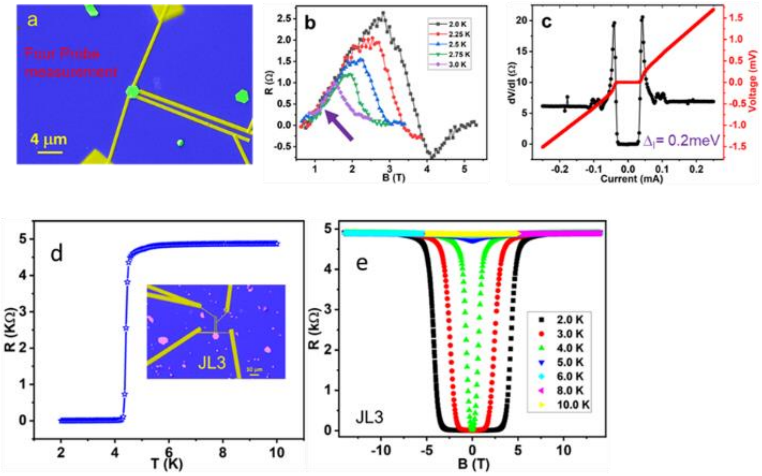

Figure S5: (a) FESEM image of Bi_2Te_3 nanosheet contacted with four W electrodes, (b) 4-probe MR curves show consistent hump features indicating Bose metal phase; (c) differential resistance (black) and IV curve (red) represent the induced superconducting gap extracted as Δ_induced ≈0.207 meV. (d) RT transport data of the JL3 device, inset shows the device image, e) represents the MR measurement of device JL3 showing absence of hump features.

Figures (a-c) present four-probe measurements performed on selected Bi_2Te_3 nanosheets. The results corroborate the superconducting transition observed in two-probe geometry, and reveal that the metallic hump feature persists in the four-probe setup. This confirms that the observed resistance anomalies originate from the TI nanosheet and not from W electrode contributions.

6. Supplementary Section 6 : Determination of Critical Fields B* and B_{min}

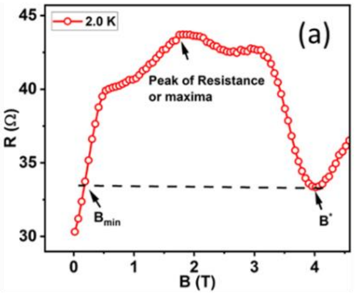

Temperature (K)	B_{min} (T)	Peak of resistance or maxima (T)	B^* (T)
2	0.178	1.87	4.015
2.25	0.176	1.86	3.563
2.5	0.170	1.77	3.12
2.75	0.166	1.59	2.66
3	0.14	1.29	2.184
3.25	0.12	1.089	1.764
3.5	0.082	0.728	1.34
3.75	0.045	0.468	0.919
4	0.02	0.244	0.522

Figure S6: The explanation of Bmin and B and estimated values can be found here. Critical field (denoted by B* in Figure (a)) is the field at which R starts to increase with decreasing field. Peak (maxima) of resistance is the cutoff between thermally assisted flux flow (TAFF) and the Bose metal state. Bmin which defines 2D Sc state is calculated by the intersection of MR curves with the black dotted line.*

7. Supplementary Section 7: Supplementary information about Bi_2Te_3 nanosheets

Synthesis: For the synthesis of Bi_2Te_3 nanostructures (hexagonal nanosheets), we employed confined thin film melting (CTFM) method as we discussed it in our earlier publications[7, 8]. Here we use a pre-deposited thin layer of Bi_2Te_3 which is having thickness less than 10 nm and used it as a source substrate. This ultra thin film was directly placed on top of the growth substrate (bare Si_3N_4) without any space in between them. The growth substrate was heated close to melting point of the Bi_2Te_3 and allowed subsequent recrystallization of the material into various nanostructures of Bi_2Te_3.

Growth Substrate: Here mostly we used silicon nitride (Si_3N_4) due to its excellent electrical insulating properties, mechanical strength and the low thermal expansion coefficient which minimizes thermal stress during the confined melting process.

Doped/Undoped: In this study, Bi_2Te_3 was synthesized under undoped conditions. Here we would like to state that there is a possibility that some Te atoms may lost due to its volatility which leaves behind Te vacancies and may release extra electrons. Since there is a large number of nanosheets, it's difficult to say that whether the nanosheet used in this study was n-doped or not since haven't performed Hall measurements on the devices reported here.

Fermi level: If the nanosheets are ultra pure-intrinsic Bi_2Te_3, the Fermi level lies in the middle of the bandgap. We used melting and annealing process during synthesis of Bi_2Te_3 nanosheet and if its n-type doping would be closer to the conduction band (assuming the intrinsic donor-like defects e.g. tellurium vacancies). This case is more favourable because during melting, Te (BP ~ 987 °C) atoms may evaporates more easily and escape as gas than Bi (BP 1564 °C).

Device Fabrication Method: A focused ion beam technique assisted by gas injection system (GIS) was used to fabricate the devices. The hexagonal Bi_2Te_3 nanosheet grown on the Si_3N_4 substrate was localized using the FESEM (Field emission scanning electron microscope). The nanosheets were never exposed to the Gallium ions. The GIS was employed to perform localized deposition of tungsten electrodes on the Bi_2Te_3 nanosheets. This enables direct writing of W electrodes down to 100-nanometer resolution.

1. Kong, D.; Dang, W.; Cha, J. J.; Li, H.; Meister, S.; Peng, H.; Liu, Z.; Cui, Y., Few-layer nanoplates of Bi2Se3 and Bi2Te3 with highly tunable chemical potential. *Nano letters* **2010,** *10* (6), 2245-2250.
2. Hao, G.; Qi, X.; Yang, L.; Liu, Y.; Li, J.; Ren, L.; Sun, F.; Zhong, J., Growth and surface potential characterization of Bi2Te3 nanoplates. *AIP Advances* **2012,** *2* (1).
3. Kong, D.; Randel, J. C.; Peng, H.; Cha, J. J.; Meister, S.; Lai, K.; Chen, Y.; Shen, Z.-X.; Manoharan, H. C.; Cui, Y., Topological insulator nanowires and nanoribbons. *Nano letters* **2010,** *10* (1), 329-333.
4. Yuan, J.; Zhao, M.; Yu, W.; Lu, Y.; Chen, C.; Xu, M.; Li, S.; Loh, K. P.; Bao, Q., Raman spectroscopy of two-dimensional Bi2Te x Se3− x platelets produced by solvothermal method. *Materials* **2015,** *8* (8), 5007-5017.

5. Wang, C.; Zhu, X.; Nilsson, L.; Wen, J.; Wang, G.; Shan, X.; Zhang, Q.; Zhang, S.; Jia, J.; Xue, Q., In situ Raman spectroscopy of topological insulator Bi_2Te_3 films with varying thickness. *Nano Research* **2013,** *6* (9), 688-692.
6. Noguchi, R.; Kobayashi, M.; Jiang, Z.; Kuroda, K.; Takahashi, T.; Xu, Z.; Lee, D.; Hirayama, M.; Ochi, M.; Shirasawa, T., Evidence for a higher-order topological insulator in a three-dimensional material built from van der Waals stacking of bismuth-halide chains. *Nature Materials* **2021,** *20* (4), 473-479.
7. Sharma, A.; Senguttuvan, T.; Ojha, V.; Husale, S., Novel synthesis of topological insulator based nanostructures (Bi_2Te_3) demonstrating high performance photodetection. *Scientific reports* **2019,** *9* (1), 3804.
8. Pandey, A.; Banerjee, S.; Yadav, R.; Kumar, S.; Jewariya, M.; Chowdhury, D. R.; Lalla, N.; Husale, S., Broadband THz absorption using nanosheets of Bi 2 Te 3 grown on a transparent conductor. *Journal of Materials Chemistry C* **2023,** *11* (4), 1448-1456.
